# Knee pain in young sports players aged 6–15 years: a cross-sectional study in Japan

**DOI:** 10.1186/s13102-022-00606-y

**Published:** 2023-02-07

**Authors:** Jun Iwatsu, Yutaka Yabe, Takuya Sekiguchi, Haruki Momma, Masahiro Tsuchiya, Kenji Kanazawa, Shinichirou Yoshida, Yasuhito Sogi, Ryoichi Nagatomi, Yoshihiro Hagiwara

**Affiliations:** 1 Department of Orthopaedic Surgery, Tohoku University School of Medicine, 1-1 Seiryo-machi, Aoba-ku, Sendai 980-8574 Japan; 2 Department of Medicine and Science in Sports and Exercise, Tohoku University School of Medicine, 2-1 Seiryo-machi, Aoba-ku, Sendai 980-8575 Japan; 3 Department of Nursing, Faculty of Health Science, Tohoku Fukushi University, 1-8-1 Kunimi, Aoba-ku, Sendai 981-8522 Japan; 4 Division of Biomedical Engineering for Health and Welfare, Tohoku University Graduate School of Biomedical Engineering, 2-1 Seiryo-machi, Aoba-ku, Sendai 980-8575 Japan

**Keywords:** Knee pain, Young sports players, Sports discipline

## Abstract

**Background:**

Knee is the most commonly injured part of the body in young athletes. Knee pain in several studies have been seen to be more prevalent in active adolescents compared to inactive, although common in both groups. Nevertheless, few studies with large sample size have been published reporting the difference of the prevalence of knee pain for each sport among young sports players. This study investigated the point prevalence of knee pain among young sports players aged 6–15 years old according to age, sex, and sports discipline. Furthermore, this study investigated the association between knee pain and sports discipline among young sports players.

**Methods:**

A cross-sectional study was conducted using a self-reported questionnaire on young sports players aged 6–15 years from the Miyagi Amateur Sports Association. Multivariable logistic regression models were used to examine the association between knee pain and sports discipline and were adjusted for age, sex, body mass index (BMI), training days per week, and training hours per weekday and weekend.

**Results:**

A total of 7234 young sports players were included. The point prevalence of knee pain was 10.9%. Females (13.3%) had more knee pain than males (9.8%). Young, 13-year-old sports players had the highest prevalence of knee pain (19.1%). The multivariable analysis showed that the highest odds ratio [95% confidence interval] was observed for handball players (2.42 [1.01–5.81]). In addition, hand ball, mini-basketball (odds ratio 1.85; 95% CI 1.38–2.47), and basketball (odds ratio 1.66; 95% CI 1.23–2.26) were significantly associated with knee pain, compared with football. The lowest odds ratio was observed for swimming (0.34 [0.05–2.54]), followed by karate (odds ratio 0.38; 95% CI 0.16–0.89) and baseball (odds ratio 0.47; 95% CI 0.35–0.64).

**Conclusion:**

The prevalence of knee pain among young athletes differed according to age, sex, and sports discipline. Their parents and clinicians should recognize this information to manage knee pain among young sports players.

**Supplementary Information:**

The online version contains supplementary material available at 10.1186/s13102-022-00606-y.

## Background

Knee is the most commonly injured part of the body in young athletes, with the prevalence of knee pain ranging from 5 to 26%, depending on sport types [1]. Acute and chronic injuries, such as osteoarthritis, patella tendinopathy, Osgood-Schlatter disease, patellofemoral pain, and infection, are the major causes of knee pain in adolescents and adults [2]. Among young athletes, a common cause of knee pain is overuse injury, including Osgood-Schlatter disease and patellofemoral pain [3, 4]. Most young athletes with knee injuries are able to return to the previous level of sports activity through conservative treatment, such as rest from painful activities, icing, and medications [5]. However, knee pain related to sports activities is commonly recurrent or chronic [6], and overuse injury in young athletes can have long-term consequences such as persistence of pain, genu recurvatum, and fragmentation of the ossicle and patella alta, which lead to early osteoarthritic changes and necessitates additional treatment [7].

Some researchers have reported risk factors for knee pain among young athletes in several sports disciplines [8–13]. Associated clinical factors for knee pain include female sex [11], older age [8, 9, 12], BMI [14], more frequent sports participation [10, 13], and type of sport [10]. Junge et al. reported risk factors for knee injuries among 1326 young athletes aged 8–15 years in nine sports [10]. Majewski et al. reported on the epidemiology of knee injuries in 6434 athletes across all age groups, including children and adolescents, among 26 types of sports [15]. However, only a small number of studies comparing knee pain among different sports disciplines in young athletes were conducted. Comparing the prevalence of knee pain among different sports is important to understand the mechanism and the motion causing knee pain. Therefore, the present study aimed to report on the point prevalence of knee pain in young athletes aged 6–15 years among 14 different types of sports and to investigate the association between knee pain and sports discipline.

## Methods

### Participants

**Fig. 1:**
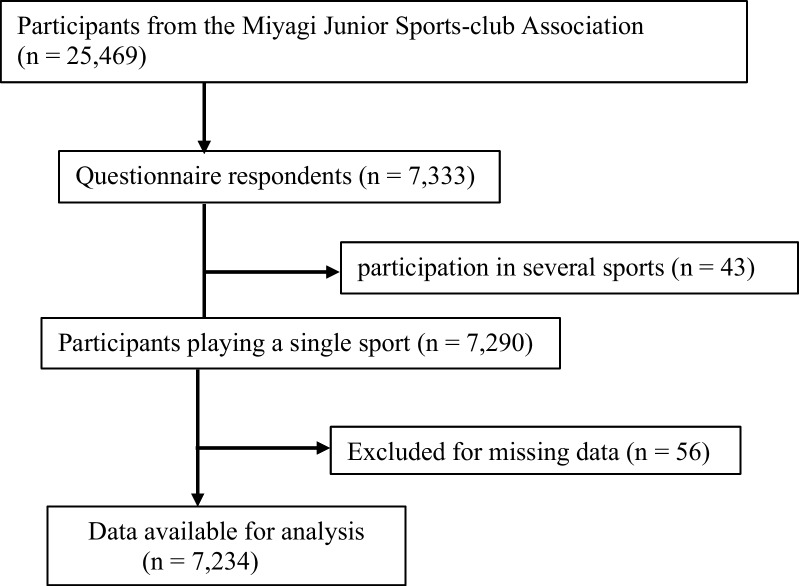
Study flow chart

A cross-sectional study was conducted on 25,469 young sports players aged 6–15 years from the Miyagi Amateur Sports Association located in Miyagi Prefecture, Japan [16]. This study was a part of an inclusive survey of young sports players to analyze their sports activities, problems, and lifestyles. A self-reported questionnaire and informed consent form were sent by mail to all the young sports players in October 2014. The participants completed the questionnaire themselves or with the help of their parents, when necessary, particularly in the case of younger participants [17]. A total of 7333 athletes (28.8%) provided consent, completed the questionnaire, and sent their answers through mail by the end of December 2014. Participants with missing age or sex data (n = 56) and those who played several sports (n = 43) were excluded from the analysis. Finally, 7234 young sports players were enrolled in this study (Fig. 1). This study was conducted in accordance with the principles embodied in the Declaration of Helsinki, and the study protocol was approved by the institutional review board of Tohoku University School of Medicine (Approval Number: 2013-564).

### Questionnaire

The questions were used to obtain data on participants’ age, sex, height, body weight, type of sport played, number of training days per week, and number of training hours per weekday and weekend. In addition, knee pain was assessed by a self-reported questionnaire. Participants were considered to have knee pain if they answered “yes” to the question which was “Do you have any pain in any parts of your body?” and specified the knee as the location of their pain by checking knee area which was illustrated by drawing (Additional file 1) [18].

### Statistical analysis

Continuous variables are presented as means and standard deviations, whereas categorical variables are expressed as numbers and percentages. The prevalence of knee pain was calculated based on age, sex, and sports discipline (the number of participants more than 30). Multivariable logistic regression models were used to examine the association between knee pain and sports discipline and were adjusted for age, sex, BMI, training days per week, and training hours per weekday and weekend. The results are presented as odds ratios (ORs) with 95% confidence intervals (CIs). Football, which is often reported as a sport that commonly causes knee pain [9, 12], was used as the reference sport in the multivariable analysis. All statistical analyses were performed using SPSS 24.0 (SPSS Japan Inc., Tokyo, Japan), with a P value of < 0.05 being considered to be indicative of statistical significance.

## Results

### Baseline characteristics

The baseline characteristics of participants are presented in Table 1. Among 7234 participants, the mean age was 10.8 years (range, 6–15 years) and the number of males and females was 5082 (70.3%) and 2152 (29.7%), respectively. Fourteen types of sports were included in this study, with baseball being the most popular sport (1748 participants), followed by football (1477 participants) (Table 1).

**Table 1:** Baseline characteristics of participants

Sports discipline	n	Age, mean (SD)	Male, n (%)	Female, n (%)	BMI, mean (SD)	Practice days per week, mean (SD)	Practice hours per day (weekdays), mean (SD)	Practice hours per day (weekends), mean (SD)
All players	7234	10.8 (1.9)	5082 (70.3)	2152 (29.7)	17.9 (2.9)	3.3 (1.5)	1.9 (1.1)	3.7 (2.1)
Baseball	1748	10.8 (1.8)	1660 (95.0)	88 (5.0)	18.1 (3.1)	3.3 (1.5)	1.5 (1.3)	5.4 (2.2)
Football	1477	10.3 (1.6)	1385 (93.8)	92 (6.2)	17.0 (2.3)	3.1 (1.1)	1.7 (0.9)	3.0 (1.2)
Mini-basketball	797	10.3 (1.5)	434 (54.5)	363 (45.5)	17.0 (2.3)	3.7 (0.9)	2.3 (0.9)	3.4 (1.5)
Volleyball	681	10.8 (1.8)	167 (24.5)	514 (75.5)	17.8 (2.5)	3.4 (1.3)	2.3 (1.4)	4.0 (2.3)
Basketball	680	12.5 (1.6)	371 (54.6)	309 (45.4)	18.5 (2.6)	4.8 (1.7)	2.2 (0.7)	3.6 (1.6)
Kendo	525	11.0 (2.2)	329 (62.7)	196 (37.3)	18.4 (3.2)	3.1 (1.2)	2.0 (0.9)	1.8 (1.2)
Judo	242	11.0 (2.3)	178 (73.6)	64 (26.4)	20.8 (4.8)	2.9 (0.9)	2.1 (0.6)	1.6 (1.1)
Karate	236	9.9 (2.2)	169 (71.6)	67 (28.4)	17.8 (2.9)	2.4 (1.1)	1.9 (0.8)	1.8 (1.5)
Track and field	137	10.3 (1.7)	57 (41.6)	80 (58.4)	17.3 (2.4)	1.7 (0.9)	0.9 (0.9)	2.1 (0.6)
Soft tennis	128	12.1 (1.9)	48 (37.5)	80 (62.5)	18.5 (2.6)	3.7 (1.9)	2.0 (0.8)	4.4 (2.5)
Badminton	123	11. 3 (1.9)	39 (31.7)	84 (68.3)	17.9 (2.5)	2.9 (1.1)	2.1 (0.7)	3.5 (1.7)
Table tennis	94	12.8 (1.9)	37 (39.4)	57 (60.6)	18.7 (2.9)	3.7 (1.9)	2.3 (0.8)	2.9 (1.8)
Handball	41	11.2 (2.3)	24 (58.5)	17 (41.5)	18.3 (2.6)	3.3 (2.0)	2.7 (0.4)	3.3 (0.8)
Swimming	41	9.9 (2.0)	29 (70.7)	12 (29.3)	18.6 (3.1)	2.4 (1.4)	1.2 (0.5)	1.0 (1.0)
Others	284	11 (2.3)	155 (54.6)	129 (45.4)	18.3 (2.9)	2.4 (1.6)	1.7 (0.8)	2.7 (2.0)

### Prevalence of knee pain according to age and sex

**Fig. 2:**
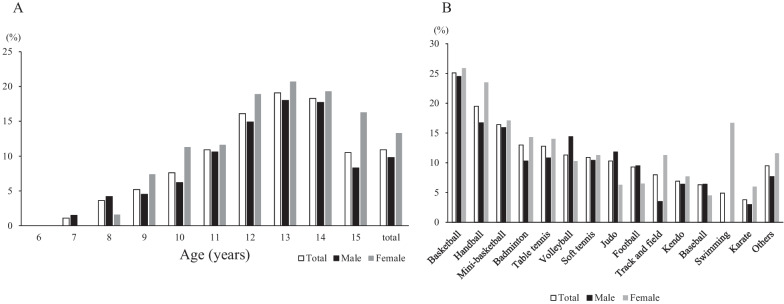
**a** The prevalence of knee pain categorized by age and sex, **b** the prevalence of knee pain categorized by sports discipline and sex

The overall point prevalence of knee pain was 10.9% (n = 787). The prevalence of knee pain was 9.8% (n = 500) among males and 13.3% (n = 287) among females. Participants aged 6 years reported no knee pain, and 19.1% of participants aged 13 years reported knee pain. In 9- and more than 9-year-old participants, the prevalence of knee pain was higher in females between 1 and 8% than in males (Fig. 2A).

### Prevalence of knee pain according to sports discipline

The prevalence of knee pain according to sports and sex is shown in Fig. 2B. Among the 14 sports, basketball players had the highest prevalence of knee pain (25.1%), followed by handball (19.5%), mini-basketball (16.4%), and badminton (13.0%) players. In males, the highest prevalence of knee pain was observed among basketball players (24.5%) followed by handball (16.7%), mini-basketball (15.9%) and volleyball (14.4%) players. In females, the highest prevalence of knee pain was observed among basketball players (25.9%), followed by handball (23.5%), mini-basketball (17.1%) players and swimmers (16.7%). The lowest prevalence of knee pain among all participants was observed among karate participants (3.8%), followed by swimming (4.9%), baseball (6.3%), and kendo (6.9%) participants. The prevalence of knee pain was higher among females than among males in all sports, except for volleyball, judo, football, and baseball.

### Association between sports discipline and knee pain

After adjustment for sex, age, BMI, training days per week, and training hours per weekday and weekend, the highest adjusted OR was observed for handball (OR 2.42; 95% CI 1.01–5.81), followed by mini-basketball (OR 1.85; 95% CI 1.38–2.47), and basketball (OR 1.66; 95% CI 1.23–2.26). With football as the reference sport, handball, mini-basketball, and basketball were identified to be significantly associated with knee pain. Furthermore, the lowest adjusted OR was observed for swimming (OR 0.34; 95% CI 0.05–2.54), followed by karate (OR 0.38; 95% CI 0.16–0.89) and baseball (OR 0.47; 95% CI 0.35–0.64) (Table 2).

**Table 2:** Association between sports discipline and knee pain in multivariate analysis

Variables	Adjusted OR (95% CI)
Football	1.00
Handball	2.42 (1.01–5.81)
Mini-basketball	1.85 (1.38–2.47)
Basketball	1.66 (1.23–2.26)
Badminton	1.23 (0.67–2.26)
Volleyball	1.08 (0.76–1.54)
Track and field	1.00 (0.48–2.07)
Judo	0.85 (0.49–1.47)
Table tennis	0.80 (0.40–1.60)
Soft tennis	0.76 (0.40–1.43)
Kendo	0.62 (0.40–0.96)
Baseball	0.47 (0.35–0.64)
Karate	0.38 (0.16–0.89)
Swimming	0.34 (0.05–2.54)
Others	1.23 (0.75–2.01)

Adjusted by sex, age, BMI, training days per week, training hours per weekday, and training hours per weekend

*OR* odds ratio, *CI* confidential interval, *BMI* body mass index

## Discussion

We examined knee pain in 7234 young sports players aged 6–15 years who participated in 14 different sports. The most important finding of this study was that the prevalence of knee pain was different according to age, sex, and type of sport. The 13-year-old athletes had the highest rate of knee pain. In 9- or more than 9-year-old females, knee pain was more than that in males. Among the different sports disciplines, the highest and lowest odds ratios were observed in handball players and swimmers, respectively. Handball, mini-basketball, and basketball were significantly associated with higher rates of knee pain compared with football in the multivariable analysis.

### The prevalence of knee pain among young sports players

There have been few reports showing the prevalence of knee pain among young sports players with large sample size [9, 19]. Hall et al. reported the point prevalence of patellofemoral pain (28%) among 546 female basketball, soccer, and volleyball players in middle schools and high schools [19]. Iwame et al. reported the point prevalence of knee pain (29.4%) among 602 boy soccer players aged 8–12 years [9]. In this study, the point prevalence of knee pain among 7234 young athletes aged 6–15 years was 10.9% (n = 787), which was lower than that reported by previous studies. The difference in age distribution, intensity of sports activity, and methodology of the study might have influenced these results. Furthermore, none of the participants aged 6 years had knee pain, which can be one of the main reasons for the low ≥ rate of knee pain in our study population.

### The prevalence of knee pain according to age

Some researchers have reported that the prevalence of knee pain increases with age [8, 9, 12]. Vähäsarja et al. reported that the prevalence of knee pain was significantly higher in adolescents aged 14–15 years than in those aged 9–10 years [8]. Furthermore, Iwame et al. reported that young athletes aged 9 or older had significantly higher rates of knee pain than those aged less than 9 years [9]. In this study, the proportion of knee pain increased with age, and 13–year-old participants had the highest rate of knee pain. The amount of training increased with older age [20], which was reported to be a risk factor for the development of patella tendinopathy [14]. Increasing the amount of training might cause knee pain with older age. Furthermore, Wild et al. showed that the growth spurt during adolescence caused musculoskeletal changes, which increased anterior cruciate ligament injuries [21]. The highest rate of knee pain observed among athletes aged 13 years in this study might be associated with this growth spurt.

### The prevalence of knee pain according to sex

Many researchers have reported a higher proportion of knee pain in females than male [11, 22, 23]. Similar findings were observed in our study among all players. Differences in hip muscle strength [24] and knee joint alignment [25, 26] between males and females are known to influence the incidence of knee pain. In addition, unfavorable changes in body composition and BMI might influence the knee injury in young females [27, 28]. Ford et al. reported that the knee abduction angle was greater in young female athletes than in males, which was associated with higher rates of anterior cruciate ligament injury in females [25]. On the other hand, several studies reported that knee abduction angle might not be associated with knee pain [29, 30]. Almeida et al. described that the knee abduction angle did not have any relationship with the severity of patellofemoral pain and hip abductor peak strength [29]. Yang et al. reported that among high school athletes, males had a higher proportion of knee injuries than females [31]. In this study, with respect to sports discipline, males had a higher prevalence of knee pain than females who played volleyball, judo, football and baseball. These results indicate that knee pain can vary according to sex and sports discipline.

### The association between sports discipline and knee pain

The association between sports discipline and knee pain has also been reported in previous studies with small number of types of sports [9, 10]. Lian et al. reported that the prevalence of patella tendinopathy was the highest among elite athletes of volleyball and basketball in a study of nine sports disciplines [32]. Junge et al. reported that participation in football, handball, basketball, and rhythmic and tumbling gymnastics were risk factors for overuse knee injuries in children aged 8–15 years in nine types of sport [10]. The prevalence of knee pain was different among 14 different sports and the adjusted OR was the highest for handball, followed by mini-basketball, and basketball in this study. Handball, mini-basketball and basketball were significantly associated with knee pain compared to football. Basketball and handball players repeatedly jump and shoot with arm swing and changes in their movement in all directions [33–35]. On the other hand, the adjusted OR was the lowest for swimming, followed by karate and baseball in this study. The specific movements and different mechanisms underlying knee injury for each sport and player function may result in different knee pain prevalence rates [36, 37], which should be clarified in future studies.

### Clinical implications

Parents or clinicians recognizing this information helped in the early diagnosis and treatment initiation, which was needed because the early management of knee pain in adolescent athletes could prevent from worsening of functional limitations [38] as well as the quality of life [39]. Furthermore, parents or clinicians should consider how to prevent child and adolescents from developing knee pain associated with exercises. Achenbach et al. reported that the frequent neuromuscular exercise could prevent adolescent handball players from severe knee injury [40].

### Limitations

The present study had some limitations. First, the response rate was not high, and only 28.8% of athletes responded to the questionnaire. Further, this study did not estimate the sample size. Second, the characteristics of knee pain, such as pain intensity, duration, location, and time of onset, were not assessed. Further, other factors such as psychological, hormonal, or sociological factors might influence knee pain [41, 42]; however, such factors were not considered in this study. Third, this study had no control group in which participants who did not participate in sports had knee pain. Finally, participants were not longitudinally observed, and knee pain that was not confirmed to have occurred as a result of sport might have been due to other causes unrelated to sport.

## Conclusions

Based on this study, the prevalence of knee pain among young sports players differed according to age, sex, and sports discipline. Especially, handball, basketball, and mini-basketball were associated with knee pain. Their parents and clinicians should recognize this information to prevent and to diagnosis and treat knee pain among young sports players.

## Supplementary Information

**Additional file 1.** Questionnaire of this study. We have collected the information about age, sex, height, body weight, type of sport played, number of training days per week, and number of training hours per weekday and weekend from 7 questions.

## Data Availability

The datasets used and/or analyzed during the current study are not publicly available due to including personal information but are available from the corresponding author on reasonable request.

## Abbreviations

OR: Odds ratio
CI: Confidential interval
BMI: Body mass index
