# Supplementary material for: Knee pain in young sports players aged 6–15 years: a cross-sectional study in Japan
Source: BMC Sports Sci Med Rehabil. 2023 Feb 7;15:16. doi: 10.1186/s13102-022-00606-y (PMC9906902; doi:10.1186/s13102-022-00606-y)
Supplement: Supplementary file 1 — Additional file 1. Questionnaire of this study. We have collected the information about age, sex, height, body weight, type of sport played, number of training days per week, and number of training hours per weekday and weekend from 7 questions. [file 13102_2022_606_MOESM1_ESM.docx]

Q. How old are you?

__ years old.

Q. Which is your gender?

Male/ female

Q. Please tell us your current height and weight.

Height __ cm, weight __ kg

Q. What type of sports are you participating in the sports club association?

Baseball, football, volleyball, mini-basketball, basketball, judo, kendo, karate, softball, handball, tennis, track and field, skiing, soft-tennis, table tennis, badminton, others.

Q. How many days a week do you participate in the sports club association?

__ days a week

Q. How many hours a day do you participate in the sports club association on weekdays and weekends?

Weekday __ hours a day

Weekends __ hours a day

Q. Do you have any pain in any parts of your body?

Yes/ No

If yes, where does it hurt? Please circle all the painful areas.


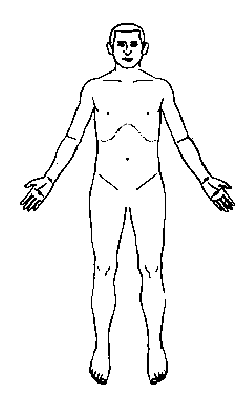


Head

Face

Left shoulder

Left elbow

Left hand

Left knee

Left hip

Left foot

Right foot

Right knee

Right hip

Right hand

Right elbow

Right shoulder


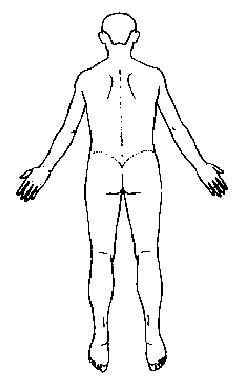


Back

Right buttock

Left buttock

Lower back
